# Functional roles of immune cells in osteoporosis

**DOI:** 10.3389/fimmu.2025.1698283

**Published:** 2025-11-24

**Authors:** Zhenqian Qi, Jiayi Luo, Zheng Xiao, Dazhi Yang

**Affiliations:** 1 Faculty of Medicine, Shenzhen University, Shenzhen, Guangdong, China; 2 Department of Spinal Surgery, The Sixth People’s Hospital of Shenzhen, Shenzhen, Guangdong, China; 3 Department of Orthopedics, Aviation Industry Corporation of China (AICC) 363 Hospital, Chengdu, Sichuan, China

**Keywords:** osteoporosis, cytokines, bone remodeling, osteoimmunology, T cells, macrophages

## Abstract

Osteoporosis is a systemic skeletal disorder characterized by reduced bone mass and deterioration of the bone microarchitecture, resulting in an increased risk of fragility fractures. Emerging evidence underscores the crucial role of immune cells as central regulators of bone metabolism. Various immune cells, including T lymphocytes and their subsets, such as TH1, TH2, TH17, and Treg cells, as well as B lymphocytes, macrophages, dendritic cells, neutrophils, mast cells, and eosinophils, orchestrate bone remodeling through complex mechanisms. These mechanisms include direct and indirect regulation of osteoclast differentiation and osteoblast function, often mediated by cytokine networks. For example, T-cell subsets exert diverse and sometimes opposing effects, whereas B cells modulate the RANKL/OPG axis. Macrophages exhibit a biphasic role, with pro-inflammatory M1 and anti-inflammatory M2 phenotypes differentially influencing bone homeostasis. This review synthesizes current knowledge on the functional contributions of immune cells to osteoporosis pathogenesis, highlighting their therapeutic potential for innovative treatment strategies.

## 1 Introduction

Osteoporosis is a pervasive systemic disease defined by diminished bone mass and microarchitectural deterioration of bone tissue, leading to enhanced bone fragility and fracture risk (1). Although postmenopausal estrogen deficiency remains a primary driver of bone loss, the immune system has emerged as a critical modulator of bone metabolism (2). Immune cells within the skeletal microenvironment engage in multifaceted crosstalk with bone cells, profoundly impacting the delicate balance between osteoblast-mediated bone formation and osteoclast-driven bone resorption (2).

**Figure 1:**
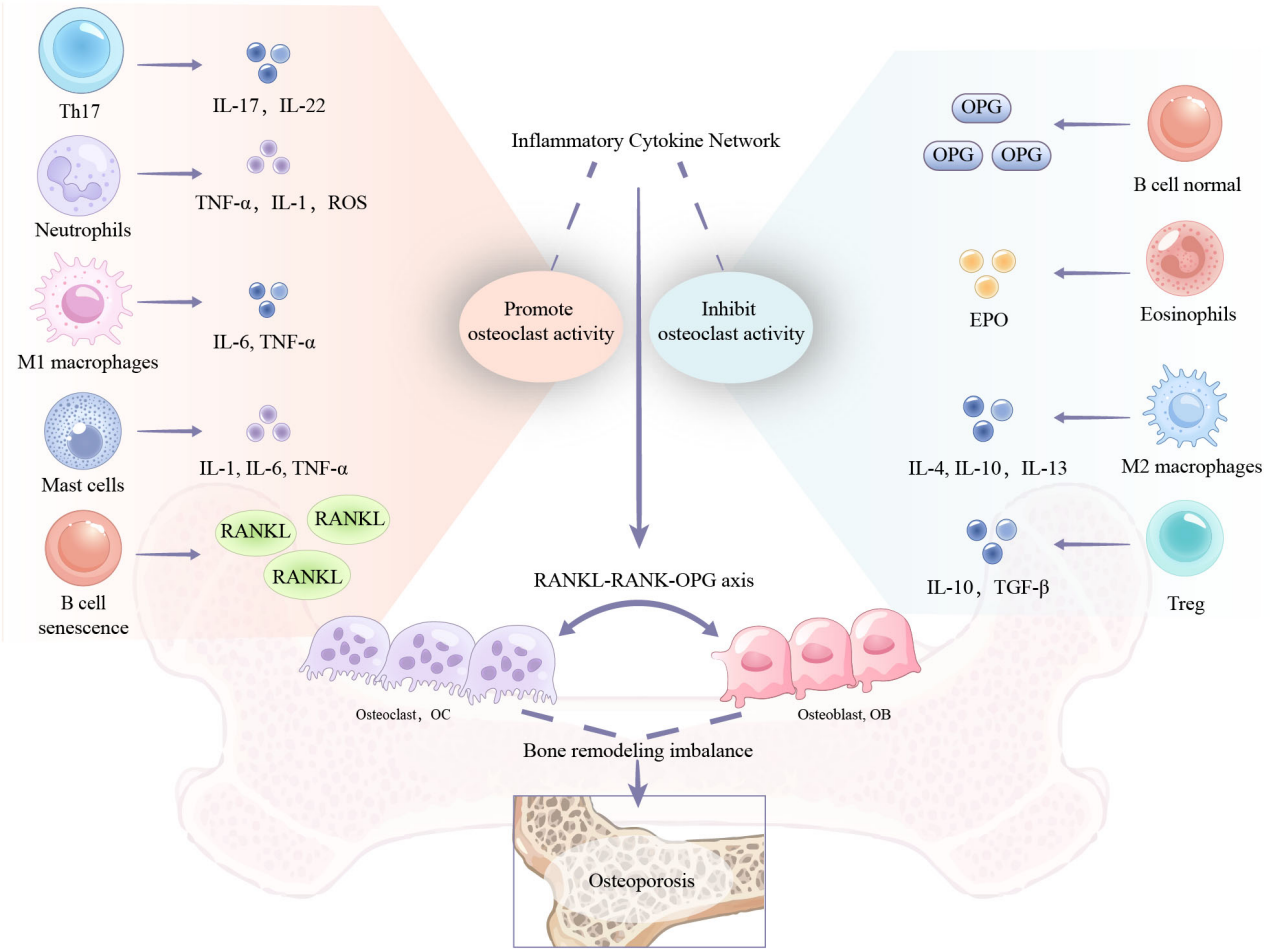
Mechanisms of immune cell regulation of bone remodeling.

In osteoporosis, immune cells contribute to pathological processes through direct and indirect mechanisms. T lymphocytes (T cells) exacerbate postmenopausal osteoporosis by secreting specific cytokines that promote osteoclastogenesis and induce osteoblast apoptosis (3). Under inflammatory conditions, activated B lymphocytes (B cells) enhance osteoclast formation through increased expression of the receptor activator of nuclear factor kappa-B ligand (RANKL) (4). Neutrophils (NE) can also potentiate osteoclastogenesis, whereas mast cells (MC) release granules rich in osteoclastogenic mediators like interleukin (IL)-6 and tumor necrosis factor-α (TNF-α) (5–7). Macrophage-derived TNF-α not only stimulates osteoclastogenesis but also inhibits osteoblast differentiation (6–9). Dendritic cells (DC) and eosinophils (EOS) have also been implicated as potential participants in the pathophysiology of osteoporosis(**Figure 1**).

This review aims to comprehensively summarize the roles of various types of immune cells in osteoporosis, elucidate their interrelationships, and discuss their integrated impact on disease progression.

## 2 Functions of immune cells in osteoporosis)

### 2.1 T lymphocytes and osteoporosis

**Figure 2:**
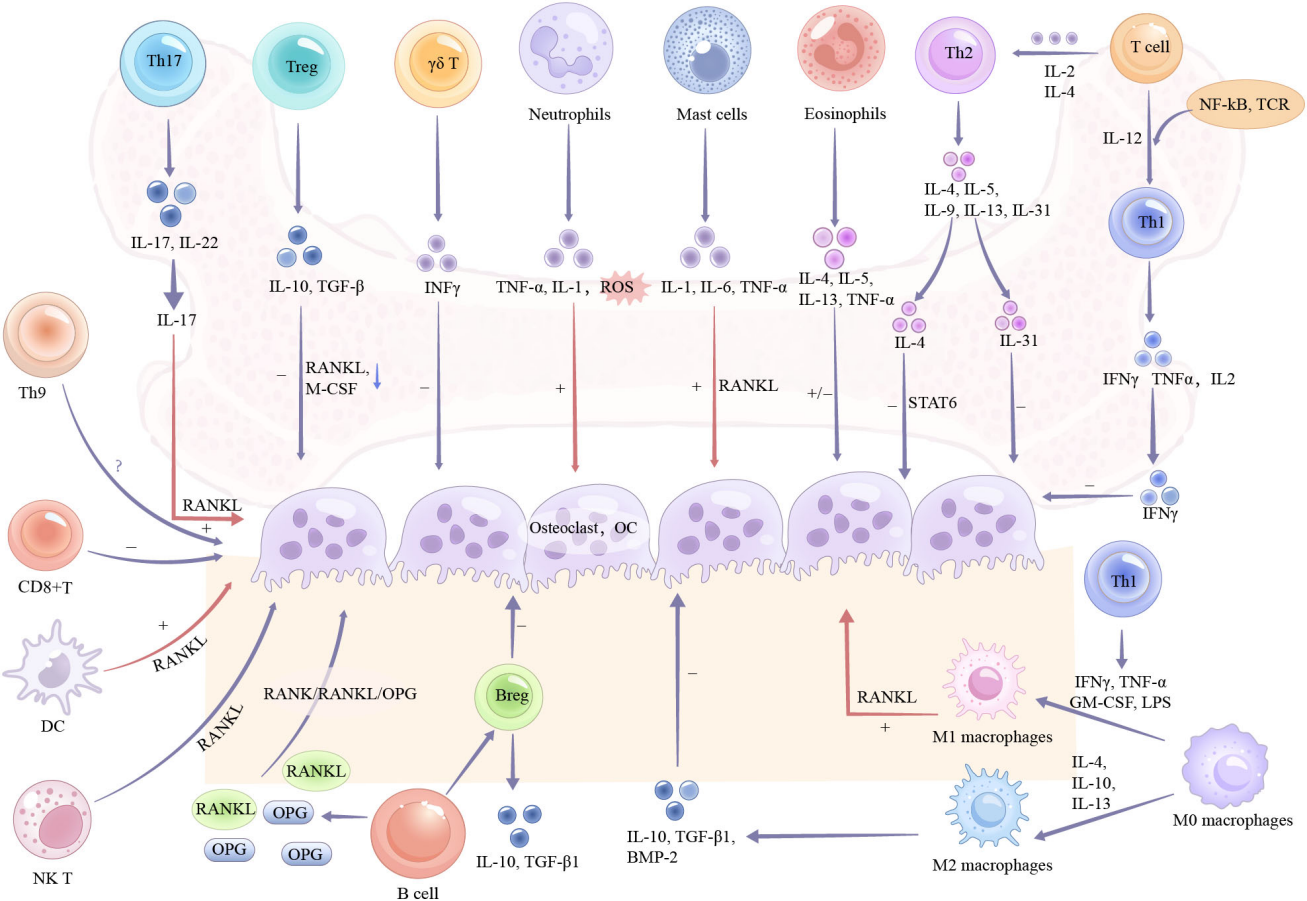
The mechanism of action between immune cells and osteoclasts.

Diverse subpopulations of T lymphocytes influence the onset and progression of osteoporosis through different mechanisms (**Figure 2**). Developed from hematopoietic stem cells and maturing in the thymus, T cells differentiate into specialized subsets with unique surface receptors and functions. CD4+ T helper cells are essential for maintaining immune system integrity, promoting B cell-mediated antibody production, and regulating CD8+ cytotoxic T cells and other immune components (10). Key subsets including Th1, Th2, Th9, Th17, natural killer T cells (NKT cells), regulatory T cells (Tregs) (11–16), gamma delta T cells (γδ T cells), and CD8+ T cells, play divergent roles in osteoporosis pathogenesis(**Table 1**).

**Table 1:** Effector T-cell subsets and osteoporosis.

Cell type	Key cytokines	Overall effect on bone remodeling	Mechanisms of action
TH1	IFN-γ, TNF-α	Dual/context-dependent	Inhibition: IFN-γ promotes the degradation of TRAF6, inhibiting RANKL-induced NF-κB/JNK pathways (18, 108). Promotion: May indirectly promote resorption under specific inflammatory conditions (e.g., estrogen deficiency).
TH2	IL-4, IL-13	Primarily anti-osteoclastogenic	Inhibits RANKL expression (22) and increases OPG expression via the STAT6 pathway (23), modulating the RANKL/OPG ratio.
	IL-31, IL-33	May promote osteoporosis	Dysregulation of the IL-33/IL-31 axis is associated with disease (26).
TH9	IL-9	Pro-osteoclastogenic (putative)	Directly promotes osteoclastogenesis (29). Enhances IL-17-producing effector T-cell function (Teff) and inhibits the anti-osteoclastogenic activity of Tregs (30).
TH17	IL-17	Strongly pro-osteoclastogenic	Upregulates RANK expression in osteoclast precursors (34). Induces RANKL expression in osteoblasts via IL-17RA/Act1 signaling (35). Directly inhibits osteoblast mineralization (33).

#### 2.1.1 TH1 cells

Th1 cells modulate bone remodeling primarily through the secretion of interferon-γ (IFN-γ) and TNF-α. IFN-γ exerts a complex influence on bone. It can suppress osteoclastogenesis by promoting rapid degradation of the key adaptor protein TRAF6, thus inhibiting RANKL-induced NF-κB and JNK signaling pathways (3, 17). For example, IFN-γ (100 U/mL) induces near-complete degradation of TRAF6 within 6 hours, effectively suppressing osteoclast formation (18).

In contrast, under specific inflammatory conditions, Th1-derived IFN-γ may paradoxically promote bone resorption. Gao et al. (19) demonstrated that IFN-γ knockout (IFN-γ^-/-^) in mice partially protected from ovariectomy (OVX)-induced bone loss, resulting in only a 14% decrease in vertebral bone mineral density (BMD) compared with a 21% decrease in wild-type mice, suggesting a contributory role of IFN-γ in estrogen deficiency-induced bone loss. Thus, the role of Th1 cells in osteoporosis is context-dependent and dualistic, capable of inhibiting and promoting bone loss through direct and indirect actions of IFN-γ.

#### 2.1.2 TH2 cells

Th2 cells, differentiate from CD4+ T cells under the influence of cytokines such as IL-2 and IL-4, and secrete IL-4, IL-5, IL-10, and IL-13. Characteristic Th2 cytokines, particularly IL-4 and IL-13, are potent inhibitors of osteoclast formation (20, 21). IL-4 inhibits RANKL mRNA expression through the STAT6 signaling pathway while simultaneously increasing osteoprotegerin (OPG) mRNA expression, thus reducing osteoclast formation and activity (22). Palmqvist et al. (23) showed that IL-4 and IL-13 significantly suppressed the expression of the 1,25(OH)2-vitamin D3-stimulated RANKL protein and increased OPG levels in mouse cranial bone tissue, thus modulating the critical RANKL/OPG ratio. This inhibitory effect was abolished in STAT6-deficient mice, confirming the necessity of the pathway.

Furthermore, Th2 cells can contribute to osteoporosis via the IL-33/IL-31 axis. Studies in postmenopausal women revealed significantly elevated serum IL-31 levels (43.12 ± 6.97 pg/mL *vs*. 29.58 ± 6.09 pg/mL in healthy controls) and significantly reduced IL-33 levels (3.53 ± 2.45 pg/mL *vs*. 13.72 ± 5.39 pg/mL) in osteoporosis patients (24, 25). A recent review corroborates that dysregulation of this axis is associated with osteoporosis (26).

#### 2.1.3 TH9 cell

The relationship between Th9 cells and osteoporosis remains poorly elucidated, though a potential association has been suggested (27). Th9 cell differentiation, driven by TGF-β and IL-4, leads to IL-9 production. This pathway shares similarities with Th17 cell differentiation (TGF-β and IL-6), and plasticity between Th9 and Th17 lineages may occur under specific conditions (28).

IL-9 has been shown to promote osteoclastogenesis in individuals with rheumatoid arthritis (RA) (29). Moreover, IL-9 enhances the function of IL-17-producing effector T cells (Teff) while simultaneously inhibiting the anti-osteoclastogenic activity of regulatory T cells (Tregs). In the presence of IL-9, Teff cell proliferation increased ~1.8-fold, whereas Treg proliferation was reduced by ~50%, and Tregs lost their capacity to inhibit osteoclast formation (30). Mechanistically, Th9-derived IL-9 promotes osteoclast genesis through a dual pathway. However, the direct role of Th9 cells in the pathogenesis of osteoporosis per se remains a significant knowledge gap and requires direct investigation.

Although these findings implicate Th9 cells in promoting bone resorption, their direct role in osteoporosis requires further investigation. Although these findings implicate Th9 cells in promoting bone resorption, their direct role in osteoporosis requires further investigation.

#### 2.1.4 TH17 cell

Th17 cells and their signature cytokine IL-17 are strongly implicated in bone loss. Bhadricha et al. (31) found significantly elevated levels of IL-17 and Th17 frequency in postmenopausal women with low BMD. A systematic review concluded that estrogen deficiency promotes osteoclast genesis by upregulating Th17 cells and increasing IL-17 secretion (32).

Tyagi et al. (33) demonstrated that IL-17 treatment significantly promoted TRAP+ osteoclast formation in bone marrow cultures and inhibited osteoblast mineralization by approximately 70% over 19 days. IL-17 promotes osteoclast differentiation by upregulating RANK expression on osteoclast precursors (34). DeSelm et al. (35) further showed that IL-17 mediated bone loss via IL-17 receptor A (IL-17RA) and its adaptor protein Act1. Notably, mice deficient in either IL-17RA or Act1 were protected from OVX-induced bone loss. Mechanistically, Act1 binds to IL-17RA and promotes RANKL expression in osteoblasts, indirectly stimulating osteoclastogenesis.

Given their prominent role, Th17 cells and their associated signaling pathways represent promising therapeutic targets for osteoporosis.

#### 2.1.5 Regulatory T cells (Tregs)

Tregs play a protective role in bone homeostasis. Zhang et al. (36) reported that the proportion of Tregs was significantly lower in an osteoporosis group (3.55 ± 2.75%) compared with both a bone loss group (4.55 ± 2.83%) and a healthy control group (7.63 ± 2.42%).

Tregs inhibit osteoclastogenesis through cell contact-dependent mechanisms, such as CTLA-4-mediated reduction of RANKL expression (37), and help maintain bone mass during remodeling (38). *In vitro*, increasing the Treg/osteoclast precursor cell ratio led to a dose-dependent decrease in osteoclast generation. In an inflammatory arthritis model, Treg injection significantly increased BMD, with the fraction of trabecular bone volume (BV/TV) increasing from 7.2 ± 0.9% to 16.2 ± 1.5% (39), demonstrating their therapeutic potential.

In particular, teriparatide treatment in humans increased the relative frequency of Tregs from 1.0% at baseline to 2.4% and 3.0% after 3 and 6 months, respectively (40). This suggests that pharmacologically enhancing Treg numbers could represent a novel osteoanabolic strategy.

#### 2.1.6 The regulatory network between Treg and Th17 cells in osteoporosis

In osteoporosis, the balance between Regulatory T cells (Treg) and helper 17 cells of T (Th17) is critical for bone homeostasis. Treg cells protect bone by secreting anti-inflammatory cytokines such as IL-10 and TGF-β, which directly inhibit the formation and function of bone-resorbing osteoclasts. In contrast, Th17 cells drive bone loss primarily through the production of IL-17, a potent cytokine that stimulates the expression of RANKL, the essential signal for osteoclast genesis. The shift in balance towards Th17 cells, resulting in an increased Th17/Treg ratio, is a key immune mechanism that underlying progressive bone loss in conditions such as postmenopausal osteoporosis (41, 42).

#### 2.1.7 Natural killer T cells

NKT cells are also involved in osteoporosis (43). Single-cell RNA sequencing of lumbar vertebral tissue from osteoporosis patients revealed that a CCL4+ NKT cell subgroup promotes disease by fostering an inflammatory environment that disrupts bone homeostasis (44).

NKT cells overexpress RANKL in osteoporosis, enhancing bone resorption. The RANKL fluorescence intensity ratio was significantly higher in patients (1.39 ± 0.11) than in healthy controls (0.99 ± 0.07), a change attributed to functional alteration rather than increased cell numbers (45).

NKT cells can be classified into subsets based on cytokine production: IFN-γ producing NKT1 (pro-inflammatory), IL-4 secreting NKT2 (anti-inflammatory), IL-10 secreting NKT10 (anti-inflammatory), and IL-17 secreting NKT17 (pro-inflammatory) cells (46, 47). The abundance of NKT1 cells in the joints of RA patients (48) suggests that the activities of NKT1 and NKT17 subsets may be linked to bone damage in osteoporosis (45).Understanding the specific mechanisms of NKT cell subsets could reveal new therapeutic insights.

#### 2.1.8 Gamma delta T cells

Although direct evidence linking γδ T cells with osteoporosis is lacking, it is proposed to regulate osteoclast genesis and bone resorption.

Activated γδ T cells secrete large amounts of IFN-γ, which inhibits osteoclast differentiation and bone resorption; an effect partially reversible upon IFN-γ neutralization (49, 50). Additionally, γδ T cells indirectly suppress osteoclast genesis by altering the differentiation of immature dendritic cells (iDC) into osteoclasts. They downregulate key osteoclastogenic genes (e.g. c-Fos and ATP6V0D2) in iDC, thus reducing the resorptive capacity (51). Based on their recognized inhibitory functions, recent insights highlight a crucial dual role for γδ T cells. Specifically, a subset known as IL-17-producing γδ T (γδ T17) can be expanded under pro-inflammatory conditions, such as estrogen deficiency and aging. In direct contrast to the effects of IFN-γ, the cytokine IL-17 potently promotes osteoclast genesis by upregulating RANKL expression in stromal cells and synergizing with other pro-inflammatory factors. This mechanism positions specific γδ T cell subsets as potential drivers of bone loss in inflammatory and metabolic forms of osteoporosis (52).

These mechanisms position γδ T cells as potential key regulators in bone immunology, possibly by balancing bone remodeling, although their role in osteoporosis warrants further study.

#### 2.1.9 CD8+T cell

The role of CD8+ T cells in osteoporosis is not fully established. A retrospective study found a correlation between decreased CD8+ T cell numbers and reduced BMD in senile osteoporosis (53). In contrast, a mouse model study showed that administration of RANKL at low (0.125 mg/kg) and high (1 mg/kg) doses increased CD8+ T cell levels, which was associated with inhibited bone resorption. However, the high-dose effect was overshadowed by the direct potent stimulation of osteoclasts by RANKL (54). The precise function of CD8+ T cells remains to be clarified.

There are conflicting findings on the role of CD8+T cells in osteoporosis. The discrepancy may arise from the different study models: human observational studies cannot establish causation, whereas mouse models have revealed complex, dose-dependent biological effects. In mice, RANKL can increase CD8+T cells that potentially inhibit bone resorption, but this protective effect is masked at high doses of RANKL due to its overpowering direct stimulation of osteoclasts.

### 2.2 B lymphocytes and osteoporosis

B lymphocytes influence bone metabolic homeostasis by secreting cytokines that modulate osteoblast and osteoclast activity (55).

**Figure 3:**
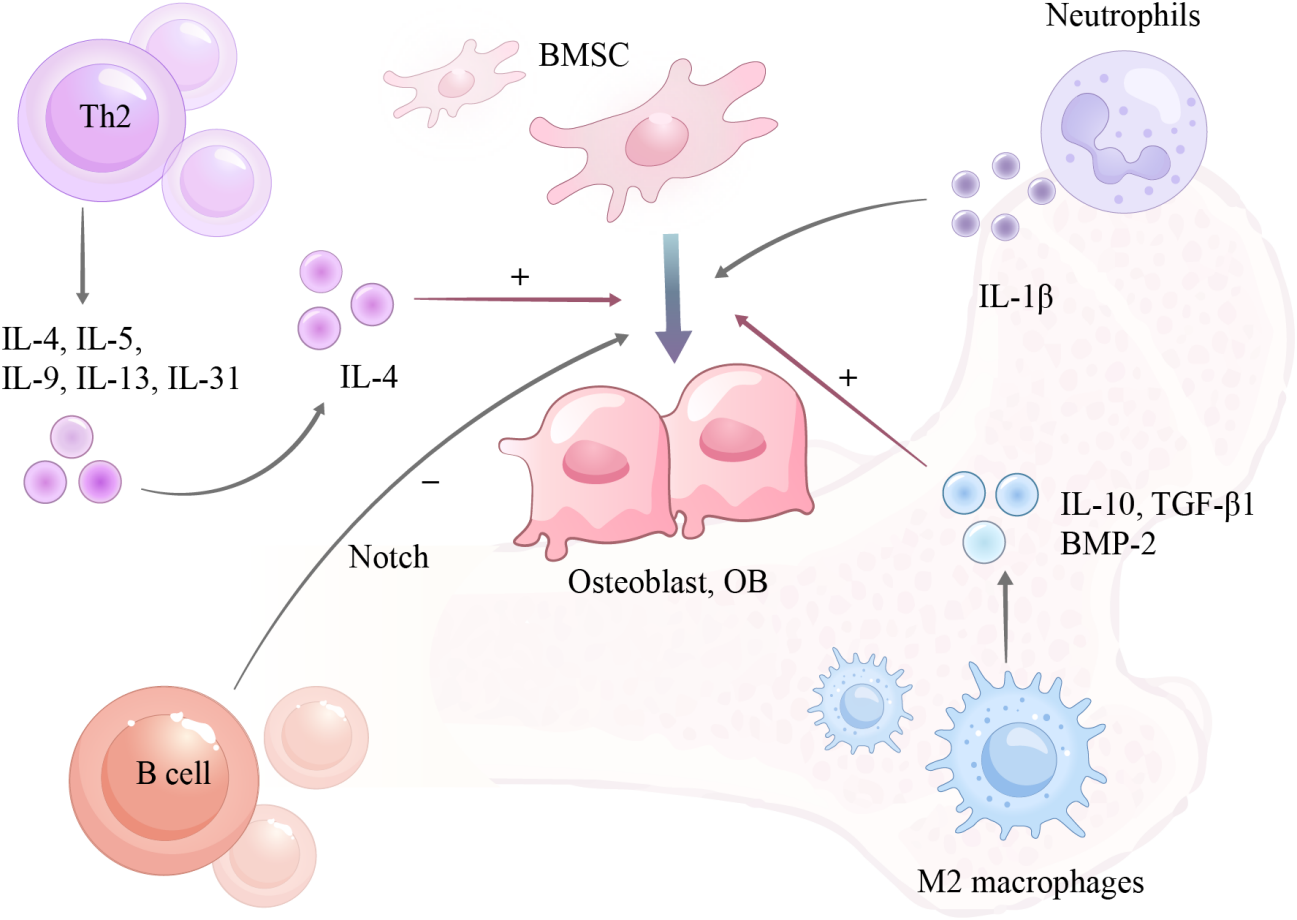
Mechanism of action between immune cells and osteoblasts.

B cells play a dual role on the RANK/RANKL/OPG axis (56, 57). In immature mice, they primarily secrete OPG, inhibiting osteoclast activity. However, with aging or inflammation, the number of B cells may decrease and their RANKL secretion increases, adjusting the balance towards resorption. Under estrogen-deficient conditions, B lymphocytes expand via CXCL12 signaling and secrete more granulocyte colony-stimulating factor (G-CSF) (58), which synergizes with RANKL to drive osteoclast proliferation and differentiation (59). Furthermore, B cells can inhibit osteoblast differentiation. They may secrete factors such as CCL3 and TNF to suppress osteogenesis (60). Lipopolysaccharide (LPS)-activated B cells inhibit osteoblast differentiation through the Notch signaling pathway, reducing the expression of the osteogenic master transcription factor RUNX2 by 51.7% and hindering BMSC osteogenic differentiation (61)(**Figure 3**).

A specialized subset, regulatory B cells (Bregs), can counteract bone loss by secreting IL-10 (62). Increasing the Breg population was associated with a decrease in Th17 cells, an increase in serum IL-10, a decrease in IL-17 and a reduction in trabecular separation in mice (from 0.27 to 0.15 mm). This confirms that Bregs inhibit osteoclastogenesis through IL-10, thus mitigating bone loss and slowing osteoporosis progression.

### 2.3 Macrophages (Mφ) and osteoporosis

Macrophages, classified primarily into the pro-inflammatory M1 and anti-inflammatory M2 phenotypes, play divergent roles in osteoporosis (63).

#### 2.3.1 M1 macrophages and osteoporosis

M1 macrophages contribute to osteoporosis by secreting pro-inflammatory cytokines such as TNF-α, IL-1, and IL-6, which promote osteoclastogenesis and/or inhibit osteoblast function. As a major source of TNF-α (64), M1 macrophages upregulate RANK expression and enhance RANKL-RANK binding efficiency, activating the NF-κB pathway and accelerating osteoclast differentiation (65). TNF-α further amplifies osteoclastogenesis via the PI3K/Akt pathway, creating a potent signaling axis (66). TNF-α also upregulates the P2Y2 receptor in mesenchymal stem cells (MSCs) by inhibiting ERK and JNK signaling, thereby inhibiting osteogenic differentiation and proliferation (67). NLRP3 inflammasome activation in macrophages leads to IL-1 release, which indirectly promotes osteoclastogenesis by stimulating prostaglandin E2 synthesis and enhancing RANKL expression in osteoblasts (68). M1-associated IL-6 promotes osteoclastogenesis and inhibits osteoblast differentiation (69). IL-6, upon binding to its soluble receptor (sIL-6R), activates gp130-mediated trans-signaling, which enhances the NF-κB and JNK pathways and enhances osteoclastogenesis induced by low concentrations of RANKL (70). IL-6/IL-6R synergy activates JAK2/STAT3 signaling, upregulating RANKL in osteoblasts; an effect abolished by the JAK2 inhibitor AG490 (71). Additionally, IL-6 affects the osteogenic capacity of bone marrow MSCs by inhibiting Wnt signaling (72, 73). Interestingly, M1 macrophages can exhibit dual functions. In certain experimental models, the anti-osteoclastogenic effects of IFN-γ and IL-12 (also secreted by macrophages of M1) can dominate the effects of pro-inflammatory factors such as TNF-α (74).

#### 2.3.2 M2 macrophages and osteoporosis

M2 macrophages, with their anti-inflammatory and reparative properties, have become a research focus for bone repair. M2 can be further subdivided into M2a, M2b, M2c, and M2d subtypes based on inducing stimuli and functions (75). IL-4 and IL-13 promote M2a polarization. M2a macrophages secrete cytokines like IL-4 and IL-13 to promote M2a polarization. M2a macrophages secrete cytokines such as BMP-2, IL-10, and TGF-β, which regulate bone matrix formation and repair. In an arthritis model, injected M2a macrophages reduced synovial inflammation and cartilage degeneration while promoting tissue regeneration (76). M2c macrophages, induced by IL-10 or glucocorticoids-induced M2c macrophages can play a key role in early wound healing by promoting matrix remodeling and phagocytosis (77). M2b macrophages, stimulated by IL-1β or immune complexes with LPS, secrete a mix of cytokines (IL-6, IL-10, TNF-α) and possess unique immunomodulatory functions. Their net effect can be context-dependent, potentially suppressing excessive inflammation but also possibly contributing to pathologies like persistent infection or tumor growth (78). M2 macrophages, particularly through IL-10 and TGF-β secretion, play a key osteogenic role. Nanoscale hydroxyapatite particles can induce M2 polarization and enhance IL-10 secretion, promoting osteogenic differentiation of MSC (79). An IL-4-loaded hydrogel scaffold further promoted BMSC osteogenesis by regulating macrophage polarization and activating the TGF-β1/Smad pathway (80). Notably, exosomes derived from M2 macrophages are enriched with osteogenic factors that significantly promote BMSC osteogenic differentiation while inhibiting adipogenesis (81, 82). Exendin-4 induced M2 polarization via the cAMP/PKA/STAT3 pathway, increasing TGF-β1 secretion, which enhanced MSC migration to bone surfaces and osteogenic differentiation (83). Combining exendin-4 with ED-71 further enhanced M2 polarization and osteogenic effects via the PI3K/AKT pathway (84).

#### 2.3.3 Crosstalk between M1 and M2 macrophages in osteoporosis

The crosstalk between M1 and M2 macrophages is a key mechanism in the pathogenesis of osteoporosis. An excess of pro-inflammatory M1 macrophages, driven by conditions such as estrogen deficiency, exacerbates bone resorption by secreting cytokines such as TNF-α, which directly enhances osteoclast formation and activity (85). Conversely, M2 macrophages counteract this process by producing anti-inflammatory mediators like IL-10 and TGF-β, which not only suppress osteoclastogenesis but also promote bone formation by stimulating osteoblast differentiation (86). Critically, this interaction is dynamic; M1-derived signals can inhibit M2 polarization, creating a vicious cycle of inflammation and bone loss. Recent single-cell RNA sequencing studies have vividly captured this imbalance, revealing a distinct expansion of M1-like macrophage subpopulations in bone marrow from osteoporotic models (87). Therefore, therapeutic strategies aimed at reprogramming macrophages from the M1 to M2 phenotype are now considered a promising frontier for restoring bone homeostasis.

### 2.4 Neutrophils and osteoporosis

The neutrophil-to-lymphocyte ratio (NLR) is associated with low BMD in postmenopausal women (88). In Chronic Obstructive lung Disease (COPD), a condition often complicated by osteoporosis, the percentage of neutrophils expressing RANKL was significantly higher in patients (6%) than in healthy controls (2%), and was even higher in patients with COPD than in those with low BMD (89). This suggests neutrophils may promote bone resorption via RANKL secretion. Chakravarti et al. (90) provided functional evidence: knocking down RANKL expression in neutrophils using antisense RNA reduced osteoclastogenesis by ~35.7% and bone resorption pit formation by ~53.9% compared with that of controls. Another study identified a TGF-β1+CCR5+ neutrophil subset associated with age-related osteoporosis in mice (91). The frequency of these neutrophils and marrow RANKL transcript levels increased with age. These neutrophils were shown to secrete TGF-β1, which promoted RANKL expression in the bone marrow, enhancing osteoclastogenesis and decreasing BMD.

### 2.5 Mast cells and osteoporosis

Mast cells, traditionally associated with allergies, have been increasingly recognized for their role in bone metabolism. The number of bone marrow mast cells was significantly higher in postmenopausal women with osteoporosis (3.38 cells/mm²) than in normal women (1.17 cells/mm²) (92). Estrogen deficiency prompts mast cells to release more cytokines (e.g., TNF-α, IL-6) that promote bone loss (93). In men with idiopathic osteoporosis, abnormal mast cell infiltrates in bone marrow biopsies have been observed, with mast cell activity correlating negatively with lumbar spine BMD (94). An *in vitro* co-culture study demonstrated that human mast cells expressing surface RANKL significantly promoted osteoclastogenesis (18.4 ± 3.9 and 20.9 ± 5.8 osteoclasts *vs*. 7.3 ± 2.7 in controls) (95)(**Figure 3**), providing direct evidence for a pro-osteoclastogenic mechanism. However, a genetic study using ovariectomized mice suggested that mast-cell-derived RANKL might be a non-critical factor in estrogen deficiency-induced bone loss (96), indicating context-dependent roles. Beyond osteoclasts, mast cells also affect osteoblasts. In systemic mastocytosis, extracellular vesicles from abnormal mast cells carry miRNAs (miR-23a, miR-30a) that enter pre-osteoblasts, inhibit their differentiation and mineralization, reduce alkaline phosphatase activity, decrease calcium deposition by > 50%, and lower RUNX2 expression (97). Thus, mast cells likely contribute to osteoporosis by dual mechanisms: promoting osteoclastogenesis and inhibiting osteoblast function. Further research is needed to clarify their specific roles in different pathological contexts.

### 2.6 Dendritic cells and osteoporosis

A link has been identified associating dendritic cells (DC) and osteoporosis, particularly in inflammatory bone loss. Alnaeeli et al. (98) demonstrated that immature CD11c+ DCs can differentiate into osteoclasts in the presence of M-CSF and RANKL, establishing DCs as a novel source of osteoclasts (DC-OCs) (99). Furthermore, DC-derived osteoclasts can stimulate T-cells to express RANKL, which in turn enhances the differentiation and activity of DC-OCs, creating a pro-resorptive feedback loop, particularly evident in RA (100). Puchner et al. (101) showed that DC depletion in an arthritis model significantly reduced joint damage and osteoclast formation, strengthening the argument that DCs are an important source of osteoclasts in inflammatory joint diseases. Although there is no direct evidence for primary osteoporosis, these findings offer valuable information to target DCs in osteoporosis treatment.

### 2.7 Eosinophils and osteoporosis

The direct link between eosinophils and osteoporosis requires further validation, but recent studies suggest a protective role. In a mouse model of collagen-induced arthritis, eosinophil injection attenuated arthritis symptoms and reduced bone erosion. Mechanistically, eosinophils promoted a shift from pro-inflammatory M1 to anti-inflammatory M2 macrophages by inhibiting the IκB and P38 MAPK pathways, thereby reducing pro-inflammatory (TNF-α, IL-6, IL-12) and increasing anti-inflammatory (TGF-β, IL-10, IL-13) cytokine levels in joints (102). Andreev et al. (103) demonstrated that eosinophils inhibit osteoclast formation and activity via their secreted peroxidase, which reduces reactive oxygen species levels in osteoclast precursors and inhibits RANKL-induced activation of the MAPK pathway (p38, JNK). *In vitro*, co-culture with eosinophils in 1:1 and 2:1 ratios reduced osteoclast numbers by 50% and 70%, respectively. These studies suggest eosinophils may help prevent inflammation-induced bone loss by modulating macrophage polarization and directly suppressing osteoclastogenesis.

### 2.8 Interaction between immune cells and osteocytes

**Figure 4:**
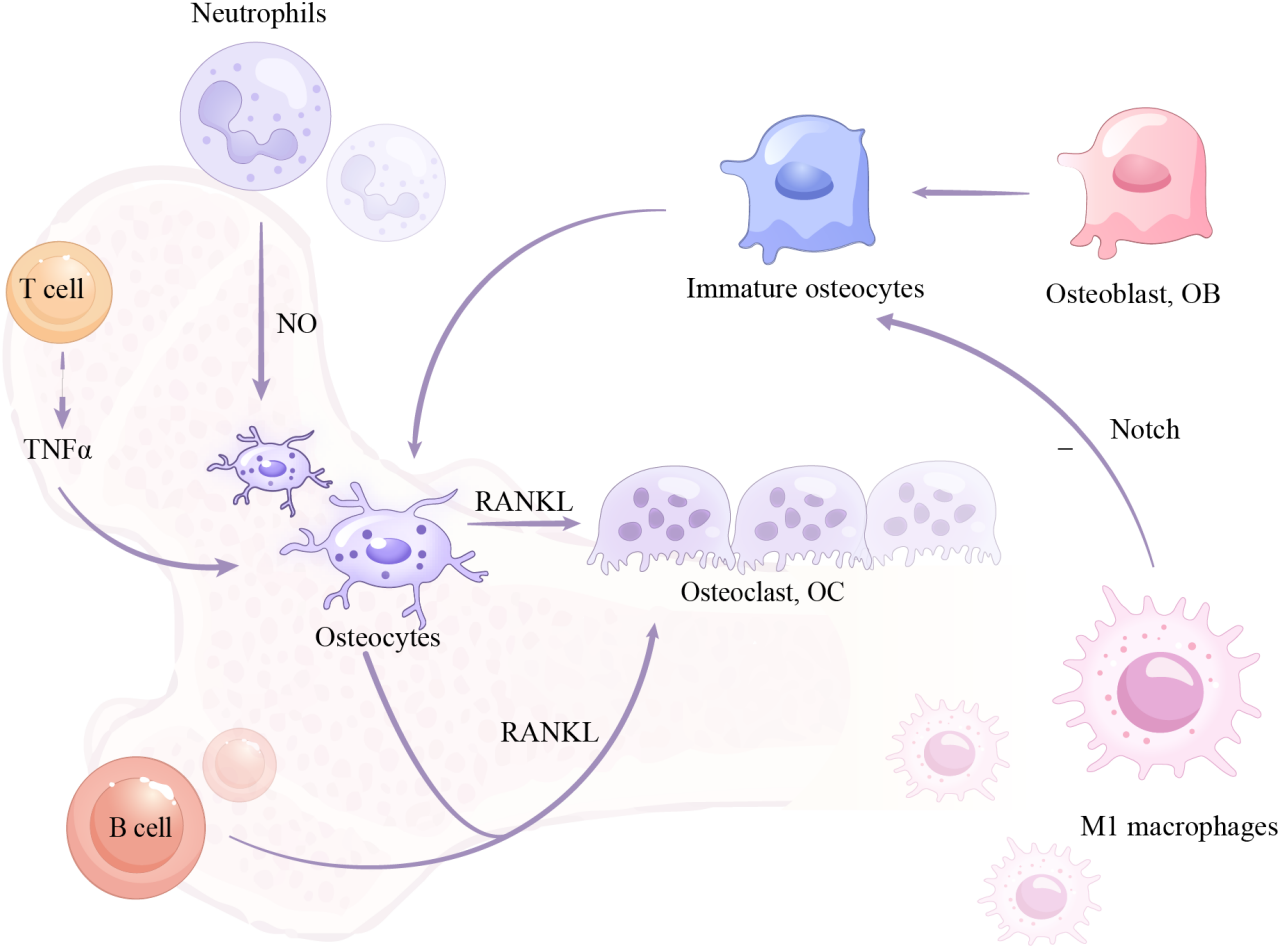
Mechanism of action between immune cells and osteoblasts.

Immune cells and osteocytes play a crucial role in bone remodeling and homeostasis. First, T cell-produced cytokines, such as TNF-α, can significantly affect osteocyte function and behavior (104). TNF-α produced by T cells binds to TNFR I and TNFR II receptors on the surface of osteocytes, leading to upregulation of RANKL expression, promoting osteoclast formation and activity, and thus absorbing bone. In the absence of estrogen, the number of B lymphocytes in the mouse bone marrow increase significantly, which is also accompanied by an increase in the number of osteoclasts and bone loss. Osteocytes are not associated with an increase in B lymphocyte number in bone marrow, indicating that osteocyte-produced RANKL is critical for these changes (105). Neutrophils secrete nitric oxide (NO), which has been shown to induce osteocyte apoptosis, and elevated NO levels are associated with increased osteocyte apoptosis, thus reducing the number of osteocytes (106). M1 macrophages can inhibit the maturation and mineralization of osteocytes by suppressing the Notch signaling pathway. Osteocyte maturation requires the activation of the Notch signaling pathway (107) and mature osteocytes produce DMP1, a crucial phosphate transporter protein essential for the mineralization of osteocytes. However, inflammatory M1 macrophages inhibit the Notch signaling pathway in osteocytes, preventing their maturation. As a result, immature osteocytes cannot produce DMP1, which negatively impacts the mineralization process of osteocytes (**Figure 4**).

## 3 Conclusion and outlook

This review provides a comprehensive overview of the pivotal role that diverse immune cells play in the pathogenesis of osteoporosis. Further, the review highlights the intricate interplay between the immune system and skeletal metabolism, extending beyond the classical paradigm of postmenopausal estrogen deficiency. We have detailed how various immune cells and their cytokine products contribute to the imbalance between bone resorption and formation. A key theme is the dualistic nature of immune regulation in bone. Specific cells and signals (e.g. Tregs, Bregs, Th2 cytokines, M2 macrophages, eosinophils) are protective, whereas others (e.g. Th17 cells, M1 macrophages, RANKL+ neutrophils, and mast cells) are destructive. The specific role of many cells (e.g. Th1, Th9, γδ T, CD8+ T cells) is highly context-dependent. A deeper understanding of the molecular mechanisms that control immune cell-bone cell interactions is crucial. Future research should prioritize exploring these mechanisms as potential therapeutic targets. Strategies could include targeting specific cytokines (e.g. IL-17), modulating the balance of the T-cell and B-cell subsets, or promoting a pro-reparative macrophage phenotype of M2. Investigating the role of novel players such as extracellular vesicles and the complex crosstalk within the immune microenvironment of bone will reveal new frontiers for the management of osteoporosis. Ultimately, translating knowledge of osteoimmunology into clinical practice holds great promise for developing innovative treatments that could significantly improve the quality of life of individuals with osteoporosis.

Building upon the foundational knowledge summarized in this review, several promising yet challenging avenues for future research emerge. A primary focus should be the precise elucidation of context-dependent roles for immune cells such as Th1, Th9, γδ T, and CD8+ T cells, whose dualistic functions are shaped by the specific bone microenvironment. The direct involvement of putative players like Th9 cells in osteoporosis pathogenesis warrants confirmation through targeted *in vivo* models. Furthermore, delving deeper into the molecular mechanisms of cellular crosstalk, particularly the role of novel mediators like extracellular vesicles and the signals governing the dynamic balance between Treg/Th17 and M1/M2 macrophages, will be crucial. Leveraging advanced technologies like single-cell and spatial transcriptomics will provide an unprecedented, high-resolution view of the osteoimmunological landscape in disease states. Ultimately, translating these mechanistic insights is paramount. Future efforts must prioritize the development of targeted immunomodulatory strategies, such as neutralizing specific cytokines (e.g., IL-17), reprogramming macrophage polarization, or restoring protective lymphocyte subsets, to pave the way for novel and precise therapeutic interventions in osteoporosis.
